# Joint UAVs’ Load Balancing and UEs’ Data Rate Fairness Optimization by Diffusion UAV Deployment Algorithm in Multi-UAV Networks

**DOI:** 10.3390/e23111470

**Published:** 2021-11-07

**Authors:** Zhirong Luan, Hongtao Jia, Ping Wang, Rong Jia, Badong Chen

**Affiliations:** 1 School of Electrical Engineering, Xi’an University of Technology, Xi’an 710021, China; 2201921137@stu.xaut.edu.cn (H.J.); 2190421160@stu.xaut.edu.cn (P.W.); jiarong@xaut.edu.cn (R.J.); 2 School of Electronic and Information Engineering, Xi’an Jiaotong University, Xi’an 710049, China; chenbd@mail.xjtu.edu.cn

**Keywords:** UAV deployment, load balancing, virtual force field, diffusion strategy, success convex approximation

## Abstract

Unmanned aerial vehicles (UAVs) can be deployed as base stations (BSs) for emergency communications of user equipments (UEs) in 5G/6G networks. In multi-UAV communication networks, UAVs’ load balancing and UEs’ data rate fairness are two challenging problems and can be optimized by UAV deployment strategies. In this work, we found that these two problems are related by the same performance metric, which makes it possible to optimize the two problems simultaneously. To solve this joint optimization problem, we propose a UAV diffusion deployment algorithm based on the virtual force field method. Firstly, according to the unique performance metric, we define two new virtual forces, which are the UAV-UAV force and UE-UAV force defined by FU and FV, respectively. FV is the main contributor to load balancing and UEs’ data rate fairness, and FU contributes to fine tuning the UEs’ data rate fairness performance. Secondly, we propose a diffusion control stratedy to the update UAV-UAV force, which optimizes FV in a distributed manner. In this diffusion strategy, each UAV optimizes the local parameter by exchanging information with neighbor UAVs, which achieve global load balancing in a distributed manner. Thirdly, we adopt the successive convex optimization method to update FU, which is a non-convex problem. The resultant force of FV and FU is used to control the UAVs’ motion. Simulation results show that the proposed algorithm outperforms the baseline algorithm on UAVs’ load balancing and UEs’ data rate fairness.

## 1. Introduction

By equipping wireless access network technology, Unmanned Aerial Vehicles (UAVs) can be used as aerial BSs to serve the ground user equipment (UE) beyond the coverage of ground BS [1,2,3]. Due to the flying nature, UAVs can be flexibly deployed to the on-demand communication areas and adapt their positions according to the communication requirements [4]. Since the UAVs operate at a high altitude, line-of-sight (LoS) links dominate the UAV-UE channel, which brings higher channel capacity. Thus, it is attractive to deploy UAVs in hotspots or disaster relief areas, where the UEs cannot be associated with ground BSs due to limited communication resources or signal coverage, in order to provide more wireless communication resources and enhance the network connectivity and capacity [5].

The research on UAV communication networks can be classified into two categories. In the first category, UAVs assist ground BSs in hotspots to improve network capacity. Generally, only a single UAV is involved in the network for traffic offloading from ground cellular networks [6,7,8]. In the second category, multiple UAVs work as a team to provide efficient communication coverage without considering the ground BS [5,9,10,11]. Considering that the BSs may not work normally in the disaster relief areas, the second scenario is more meaningful for emergency communication. Thus, we will study UAV communications in the second scenario.

One of the key factors that affect multi-UAV wireless networks’ performance is the fair coverage of UAVs. In our view, the fair coverage of multi-UAV networks has two meanings, which are the load balancing of multiple UAVs and the data rate fairness of UEs. The UAVs’ coverage fairness can be improved by optimizing the deployment of the UAVs, which is also known as the UAV positioning or the trajectory design.

Recently, there are inspiring studies on UAV deployment optimization for UAVs’ fair coverage. Liu et al. [12] proposed a deep reinforcement learning-based UAV deployment algorithm for energy-efficient communication and fair UAV coverage. Chen et al. [11] also optimized the UAV networks’ efficiency and fair using mean field deep reinforcement learning. The above two methods are both centralized. However, in a large scale multi-UAV network, a centralized algorithm requires a central controller and may result in too much information exchange and high control latency. Little work has been done on distributed fair UAV coverage optimization. Liu et al. [10] studied the distributed methods on UAVs’ motion control for long term coverage. Most of the above-related work studied one aspect of fair coverage, which is load balancing of UAVs or fairness of UEs’ data rate.

The virtual force field is an efficient tool for UAV deployment optimization due to its low computation complexity. Zhao et al. developed a distributed UAV deployment algorithm considering UAVs’ load and UEs’ data rate at the same time [5]. In that work, by considering the users’ center and the connectivity between UAVs, the positions of UAVs are controlled by the synthesis of four virtual forces. These virtual forces are based on the UEs’ distribution, UAVs’ distribution and distances between UAVs. It can be seen that the virtual force-based UAV deployment algorithm is practical due to its low computation complexity. However, these virtual forces are not constructed by any performance matrices. Such work achieves some level of fair coverage, but the performance can still be further improved. In this work, we will adopt the virtual force field method to optimize the UAVs’ deployment for fair coverage. The innovation of this work on the virtual force field method is that we define two new virtual forces by performance metric.

The load of a UAV is determined by its serving UEs. UEs cannot be associated with UAVs too far away. Thus, for any UAV in a multi-UAV network, the load can only be balanced between neighbor UAVs. So, distributed load balancing algorithms are preferred in multi-UAV networks. In this work, we consider adopting the diffusion strategy in load balancing optimization. The diffusion strategy is a new distributed method that estimates local parameters of interest through information exchange with neighbor nodes in the network [13]. Diffusion LMS (Least Mean Square) has been successfully applied in many signal processing problems [14]. The diffusion strategy is distributed and responds in real time, which makes it fit for the UAV deployment problem [15]. The diffusion LMS aims to achieve the same parameter for all nodes. However, in our problem, load balancing between UAVs does not mean the same load between UAVs. Thus, we need to modify the diffusion strategy with a new load balancing cost function and optimization process.

In this paper, we focus on joint UAV load balancing and UE data rate fairness optimization in multi-UAV networks. To this end, we adopt the virtual force method to control UAVs’ movement [5,16]. We propose a unified performance metric for both the UAV load balancing and the UE data rate fairness problem. Meanwhile, based on the unified metric, we define two new virtual forces, the namely UAV-UAV force and UE-UAV force, which are denoted by FV and FU, respectively. The goal of FV is to balance the load among UAVs. A diffusion strategy is designed to update FV, in which each UAV optimizes its FV automatically through the local information (UEs’ data rate and UAV’s load) and the load information of neighbor UAVs. Meanwhile, FU aims to achieve proportional fairness for the UEs served by one UAV, which increases the lowest data rate in the network. The optimal solution of FU is searched by the success convex optimization. The resultant force of FV and FU results in load balancing among UAVs and fairness among UEs. Since FU only exists between the UE and its serving UAV, compared with FV, the range of FU is very short. In this way, FV determines the large-scale movement of the UAVs and FU determines the fine-tuning of the UAVs’ movement. Thus, FU is the main contributor to the performance. The main contribution of this paper include:

1. A unified performance metric for joint UAVs’ load balancing and UEs’ data rate fairness optimization.
2. Construction of the virtual forces by the unified performance metric, instead of the simple location information of UEs and UAVs in [5].
3. A diffusion UAV-UAV virtual force update method and searching of the optimal UAV-UE virtual force solution by successive convex optimization.

The rest of this paper is organized as follows. We give the system model in Section 2. The proposed diffusion UAV deployment optimization algorithm is introduced in Section 3. Simulation results are presented in Section 4. Finally, Section 5 concludes this research.

## 2. System Model

### 2.1. Network Model

**Figure 1:**
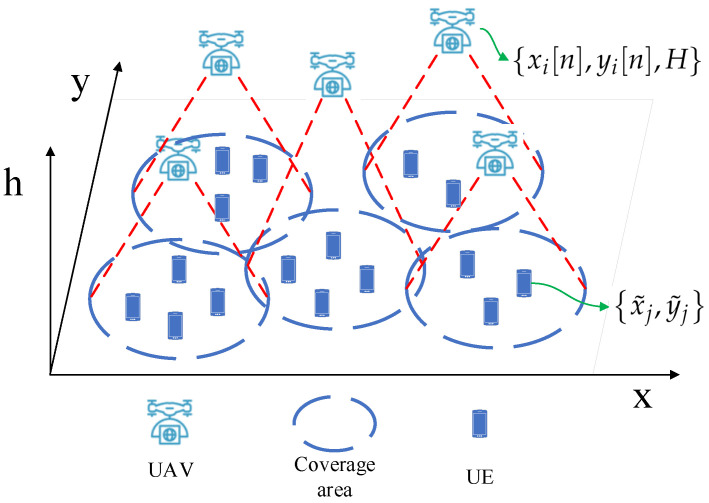
Multi-UAV communication network.

We consider a multi-UAV downlink network consisting of *N* UEs and *M* UAVs, which are denoted by $\left\{UE_{1},UE_{2},\ldots ,UE_{N}\right\}$ and $\left\{UAV_{1},UAV_{2},\ldots ,UAV_{M}\right\}$, respectively, as shown in Figure 1. The UAVs serve the ground UEs as BSs at a fixed altitude [10]. Each UE is associated with the closest UAV. The UAVs can move to any position in the area of interest. The position of $UAV_{i}$ at time *n* is denoted by $\left\{x_{i}\left[n\right],y_{i}\left[n\right],H\right\}$, where $x_{i}$[n], $y_{i}$[n] are the x- and y-horizontal coordinates at time *n* and *H* is the fixed altitude. Since the UAVs move much faster than UE, we assume that the positions of UEs are fixed in this scenario. The position of $UE_{j}$ is denoted by $\left\{{\tilde{x}}_{j},{\tilde{y}}_{j}\right\}$. The distance between $UAV_{i}$ and $UE_{j}$ at time n, which is denoted by $d_{i,j}$[n], can be calculated by

(1) $$\begin{array}{c} d_{i,j}\left[n\right]=\sqrt{{\left(x_{i}\left[n\right]-{\tilde{x}}_{j}\right)}^{2}+{\left(y_{i}\left[n\right]-{\tilde{y}}_{j}\right)}^{2}+H^{2}} \end{array}$$

Assume that the maximum UAV speed is $V_{max}$, for any $UAV_{i}$, we have

$$\sqrt{{\left(x_{i}\left[n\right]-x_{i}\left[n-1\right]\right)}^{2}+{\left(y_{i}\left[n\right]-y_{i}\left[n-1\right]\right)}^{2}}\leq V_{\max}$$

The air-to-ground channel is modeled by jointly considering line-of-sight (LoS) and non-line-of-sight(NLoS) transmission with their occurrence probability [5]. The probability of having LoS transmission between $UAV_{i}$ and $UE_{j}$ at time n is given by [17]

(2) $$\begin{array}{c} p_{i,j}^{LoS}\left[n\right]=\frac{1}{1+a\exp(-b\left(\frac{180}{\pi }tan^{-1}\left(\frac{H}{r_{i,j}\left[n\right]}-a\right)\right)} \end{array}$$

where *a* and *b* are constant values that depend on the environment, $(-b\left(\frac{180}{\pi }tan^{-1}\left(\frac{H}{r_{i,j}\left[n\right]}-a\right)\right)$ is in degrees, and

(3) $$\begin{array}{c} r_{i,j}\left[n\right]=\sqrt{{\left(x_{i}\left[n\right]-{\tilde{x}}_{j}\right)}^{2}+{\left(y_{i}\left[n\right]-{\tilde{y}}_{j}\right)}^{2}} \end{array}$$

Thus, the NLoS probability is $P_{i,j}^{NLoS}\left[n\right]=1-P_{i,j}^{LoS}\left[n\right]$.

The average path loss of LoS and NLoS transmissions between $UAV_{i}$ and $UE_{j}$ at time *n* are defined by [18,19]

$$g_{i,j}^{LoS}\left[n\right]=\eta_{LoS}\times \rho_{0}\times d_{i,j}^{-2}\left[n\right],$$

$$g_{i,j}^{NLoS}\left[n\right]=\eta_{NLoS}\times \rho_{0}\times d_{i,j}^{-2}\left[n\right]$$

where $\eta_{LoS}$ and $\eta_{NLoS}$ are average additional path loss factor for LoS and NLoS transmission, respectively, $f_{c}$ is the carrier frequency. Therefore, the probabilistic mean path gain between $UAV_{i}$ and $UE_{j}$ at time *n* is

(4) $$\begin{array}{c} g_{i,j}\left[n\right]=g_{i,j}^{LoS}\left[n\right]\times P_{i,j}^{LoS}\left[n\right]+g_{i,j}^{NLoS}\left[n\right]\times P_{i,j}^{NLoS}\left[n\right] \end{array}$$

Assuming that the UAVs share the same frequency band, the UAVs interfere with each other in downlink transmission. The signal to interference plus noise ratio (SINR) is given by [19]

(5) $$\begin{array}{c} \gamma_{i,j}\left[n\right]=\frac{p_{i}\left[n\right]g_{i,j}\left[n\right]}{\sum_{k\neq i}p_{k}\left[n\right]g_{k,j}\left[n\right]+\sigma^{2}} \end{array}$$

where $p_{i}\left[n\right]$ is the downlink transmit power of $UAV_{i}$, $p_{k}\left[n\right]$ is the interference from other UAVs, and $\sigma^{2}$ is the power of the additive white Gaussian noise (AWGN) at the receiver.

We assume that each UAV equally allocates the frequency resource to its UEs [20]. Accordingly, available bandwidth for $UE_{j}$ associated with $UAV_{i}$ is expressed as $B_{i,j}\left[n\right]$ = $B_{0}$/$N_{i}\left[n\right]$, where $B_{0}$ is the available bandwidth and $N_{i}$ is the number of UEs served by $UAV_{i}$ at time *n*. Then, the data rate of $UE_{j}$ from $UAV_{i}$ is

(6) $$\begin{array}{c} T_{i,j}\left[n\right]=B_{i,j}\left[n\right]log_{2}\left(1+\gamma_{i,j}\left[n\right]\right) \end{array}$$

The number of UEs associated with $UAV_{j}$ determines the available bandwidth of each UE.

### 2.2. Utility Formulation and Problem Defination

There are two sub-problems in this work, which are the UAVs’ load balancing and the UEs’ data rate fairness. We formulate the UEs’ data rate fairness metric by the proportional fairness as follows [21].

(7) $$\begin{array}{c} U_{i}\left(T_{i}\left[n\right]\right)=\sum_{UE_{j}\in UAV_{i}}\log(T_{i,j}\left[n\right]) \end{array}$$

where $UE_{j}$ ∈ $UAV_{i}$ mean the $UE_{j}$ which is associated with $UAV_{i}$, and **T** is the set of all UE’s throughput. The logarithm function has the property of diminishing returns, which makes the logarithm function naturally achieve fairness among UEs.

Another goal of this paper is to balance the load among UAVs by distributed UAV deployment optimization. Considering the data rate of UEs, the load of a UAV is determined by not only the number of UEs associated with this UAV but also the channel states of the UEs [20]. The load balancing can be achieved by allocating more resources to the UEs with a low rate [20,22]. In this work, we will manage the radio resource through UAV deployment optimization. Other techniques, such as user association, UAV coverage expansion or transmission power control, can also be used for load balancing optimization, which are, however, beyond the scope of this paper.

From previous studies, the logarithm function with respect to UEs’ data rate can be used to represent the load of a cell [20]. The load balancing utility of the whole multi-UAV network can be defined as

(8) $$\begin{array}{c} \;U\left(T_{i}\left[n\right]\right)=\sum_{UE_{j}}\log(T_{j}\left[n\right])\;j\in \left\{1,2,...,N\right\} \end{array}$$

The difference between (7) and (8) is that (7) only considers the UEs served by one UAV and (8) considers all the UEs. Equation (8) can be further transformed as

(9) $$\begin{array}{c} \;U\left(T_{i}\left[n\right]\right)=\sum_{UAV_{i}}U_{i}\left(T_{i}\left[n\right]\right)\;i\in \left\{1,2,...,M\right\} \end{array}$$

In this way, we find that UEs’ data rate fairness can be used to formulate the UAVs’ load balancing problem. To be more specific, UEs’ data rate fairness of one UAV can be treated as the load of this UAV.

In our work, we do not use (8) or (9) as the UAVs’ load balancing metric due to its complexity. Instead, we will use (7) as a factor to construct the UAV-UAV virtual force for load balancing optimization. Thus, the UAVs’ load balancing problem and UEs’ data rate fairness problem are unified by the same metric (7). So, these two problems can be optimized at the same time.

We define the UAV deployment problem for joint UAVs’ load balancing and UEs’ data rate fairness optimization as follows.

*Problem 1*: Given the number of UAVs and UEs, how should UAVs be deployed in a distributed manner to obtain the best downlink load balancing utility among UAVs and achieve data rate fairness among UEs?

We model *problem 1* as a continous programming problem, since we care about the continuous motion control of each UAV. The optimal goal of *problem 1* is

$$\begin{array}{c} Maximize\;U\left(T_{i}\left[n\right]\right)=\sum_{UAV_{i}}U_{i}\left(T_{i}\left[n\right]\right)\;i\in \left\{1,2,...,M\right\}. \end{array}$$

We will use the Jain’s fairness index and data rate CDF (Cumulative Distribution Function) to indicate the effectiveness on UAVs’ load balancing and UEs’ data rate fairness, respectively.

## 3. Proposed Diffusion UAV Deployment Algorithm for Fair Coverage

In this section, we will present the diffusion UAV deployment algorithm for fair coverage optimization. The algorithm does not require the UEs’ locations in advance. Instead, the UEs can be discovered by UAVs through target recognition sensors. The locations of the UAVs are initialized in random places, i.e., the center of the area, which is consistent with the UAV positioning philosophy in real systems. The UAVs’ motions are controlled by a diffusion strategy in a distributed manner. This diffusion strategy requires the information of neighbor UAVs. Meanwhile, aiming at fair UEs’ performance, the UAVs’ motions are also controlled by the UEs’ data rates.

### 3.1. Basic Idea of UAV Deployment Optimization for Load Balancing

The efficient way for load balancing optimization is to provide more radio resources to low rate UEs. For example, Luan achieves load balancing in heterogeneous networks by maximizing the worst user data rate [22].

In our problem, the UAV deployment is a way of radio resource management for the UAV-UE network. The UAV deployment manages the radio resources in three aspects, which are:

(i) UE association. Each UE is always associated with the closest UAV.
(ii) The UE receiving power level and interference power level, which is determined by the distance between UAVs and UEs.
(iii) Probability of LoS and NLoS transmissions, which are calculated by (2).

**Figure 2:**
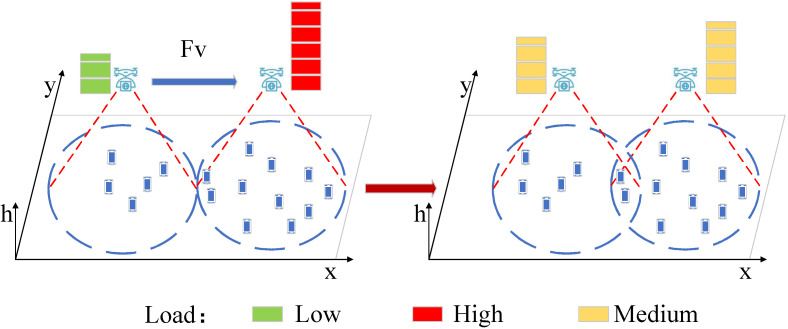
Motion of a UAV for load balancing.

Generally, the low-load UAV load should provide its radio resource to UEs that are associated with high-load neighbor UAV. By moving towards the neighbor UAV, the low-load UAV can serve more UEs. In this way, the load can be transferred to low-load UAV, which is shown in Figure 2.

**Figure 3:**
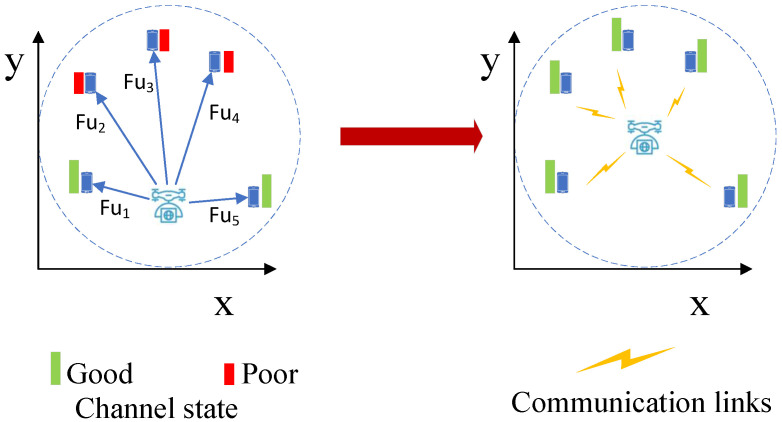
Motion of a UAV for fair coverage and higher data rate.

Meanwhile, for a signal UAV and its UEs, UAV should move to the “center” of its serving UEs to provide a fair coverage and higher data rate, which is shown in Figure 3.

From above, we have the moving trends of UAVs for load balancing. Next, we will propose the virtual force field for movement control.

### 3.2. Virtual Force Field

The virtual force field has been successfully used for UAV movement control in recent research, which is a practical heuristic method due to its low complexity [5]. In our work, we will design two virtual forces for UAV movement control, which are the UAV-UAV force and UAV-UE force, respectively, as shown in Figure 2 and Figure 3.

#### 3.2.1. UAV-UAV Force

For $UAV_{i}$, we denote the force from $UAV_{i}$ to $UAV_{k}$ by $\vec{Fv_{i,j}\left[n\right]}$. We model the UAV-UAV force as follows,

(10) $$\begin{array}{c} \vec{Fv_{i,j}\left[n\right]}=Kv\times \left(U_{i}\left(T\left[n\right]\right)-U_{k}\left(T\left[n\right]\right)\right)\times {\vec{D_{i,k}^{v}}}_{,} \end{array}$$

where *Kv* is the UAV-UAV force factor, $\vec{D_{i,k}^{v}}$ is the normalized virtual force direction vector from $UAV_{i}$ to $UAV_{k}$, which can be calculated by

(11) $$\begin{array}{c} \vec{D_{i,k}^{v}}={\frac{\left[\left(x_{k}\left[n\right]-x_{i}\left[n\right]\right)\right],\left(y_{k}\left[n\right]-y_{i}\left[n\right]\right)]}{\sqrt{{\left(x_{k}\left[n\right]-x_{i}\left[n\right]\right)}^{2}+{\left(y_{k}\left[n\right]-y_{i}\left[n\right]\right)}^{2}}}}_{.} \end{array}$$

Equation (10) consists of two parts. The first part, $Kv\times (U_{i}\left(T\left[n\right]\right)-U_{k}\left(T\left[n\right]\right))$, shows the magnitude of $\vec{Fv_{i,k}\left[n\right]}$. The second part, $\vec{D_{i,k}^{v}}$, denotes the direction of $\vec{Fv_{i,k}\left[n\right]}$. Moreover, Equation (10) can be described by a vector graphical representation as follows:

$$\begin{array}{c} \vec{Fv_{i,j}\left[n\right]}=\left(Kv\times \left(U_{i}\left(T\left[n\right]\right)-U_{k}\left(T\left[n\right]\right)\right)\right)\cdot \frac{180}{\pi }tan^{-1}\left(\frac{y_{k}\left[n\right]-y_{i}\left[n\right])}{x_{k}\left[n\right]-x_{i}\left[n\right]}\right), \end{array}$$

where the direction is in degrees.

Through load metric (7), $\vec{Fv_{i,k}\left[n\right]}$ “drags” the low load UAV to the high load UAV and “pushes” high load UAV away from low load UAV. In this way, more UEs are associated with the low load UAV, which balances the load between two UAVs.

For any two UAVs in the network, we have the following property.

**Property 1.**  *For any $UAV_{i}$ and $UAV_{k}$ in the network,*

(12) $$\begin{array}{c} \vec{Fv_{i,k}\left[n\right]}=\vec{Fv_{k,i}\left[n\right]} \end{array}$$

**Proof of Property 1.** 
$$U_{i}\left(T\left[n\right]\right)-U_{k}\left(T\left[n\right]\right)=-\left(U_{k}\left(T\left[n\right]\right)-U_{i}\left(T\left[n\right]\right)\right),$$

$$\vec{D_{i,k}^{v}}=\vec{-D_{i,k}^{v}}$$

Thus

$$\vec{Fv_{i,k}\left[n\right]}=Kv\times \left(-\left(U_{k}\left(T\left[n\right]\right)-U_{i}\left(T\left[n\right]\right)\right)\right)\times \vec{-D_{i,k}^{v}}$$

$$\vec{Fv_{i,k}\left[n\right]}=Kv\times \left(U_{k}\left(T\left[n\right]\right)-U_{i}\left(T\left[n\right]\right)\right)\times \vec{D_{i,k}^{v}}$$

$$\vec{Fv_{i,k}\left[n\right]}=\vec{Fv_{k,i}\left[n\right]}.$$

   □

This property implies that two UAVs will have the same moving direction when the load is not balanced.

#### 3.2.2. UAV-UE Force

The UAV-UE force $\vec{FU_{i}\left[n\right]}$ controls $UAV_{i}^{\prime }s$ motion for optimizing load balancing as well as data rate fairness among serving UEs of $UAV_{i}$. We define the force from $UAV_{i}$ to $UE_{j}$ by $\vec{FU_{i}\left[n\right]}$. Assume that all users have the same communication priority, the force that controls $UAV_{i}^{\prime }s$ motion is defined as the sum of UAV-UE forces from all its serving UEs, which is

(13) $$\begin{array}{c} \vec{FU_{i}\left[n\right]}=\sum_{UE_{j}\in S_{i}}\vec{Fu_{i,j}\left[n\right]} \end{array}$$

where $S_{i}$ is the set of UEs served by $UAV_{i}$.

Considering the fairness among UEs served by a same UAV, we define the UAV-UE force according to (7) as follows.

(14) $$\begin{array}{c} \vec{Fu_{i,j}\left[n\right]}=Ku\times \left(argmax_{\left\{x_{i}\left[n\right],y_{i}\left[n\right]\right\}}log\left(T_{i,j}\left[n\right]\right)-\left\{x_{i}\left[n-1\right],y_{i}\left[n-1\right]\right\}\right) \end{array}$$

s.t. (1), (2), (3), (4), (5), (6)

In (14), *Ku* is the UAV-UE force factor. It has been proven in [18] that fairness can be achieved by each UE individually optimizing his/her own logarithm of data rate.

$\vec{Fv_{i,k}\left[n\right]}$ dominates the motion of UAV as well as the load balancing among UAVs, and $\vec{Fu_{i,j}\left[n\right]}$ aims to improve the local performance. Thus, *Ku* should be bigger than *Kv* in general.

### 3.3. CTA Diffusion UAV-UAV Virtual Force Optimization

In this section, we will adopt the diffusion strategy as a decentralized tool to optimize the UAV-UAV force.

Diffusion strategy is an effective tool to optimize the global utility in a locally distributed manner [13,14,15]. The diffusion strategy updates local parameters by not only the local information but also neighbor nodes’ parameter variation trend. In this way, the coupling between nodes in the optimization process is considered. A typical Combine-then-Adapt (CTA) diffusion model consists of two steps: (1) neighbor nodes’ parameter combination, (2) local parameter update. For more details about the CTA diffusion model, please refer to [13].

**Figure 4:**
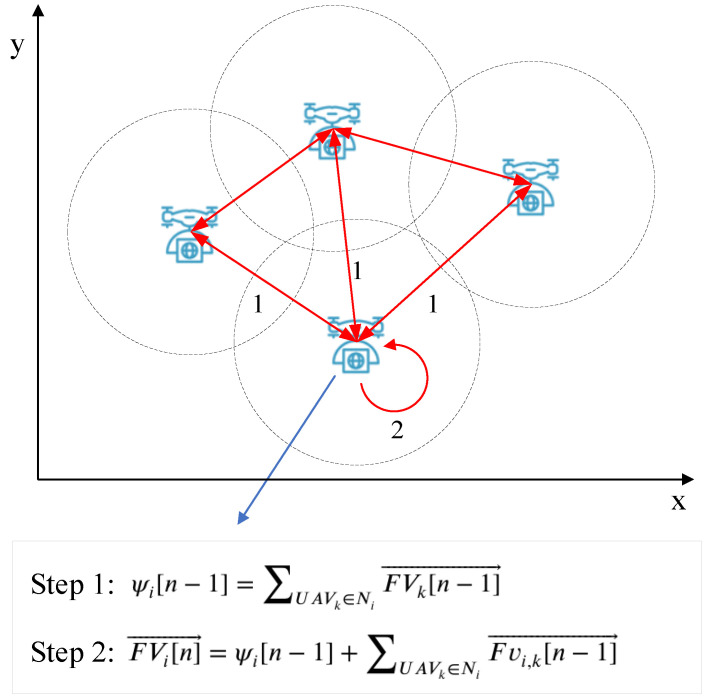
CTA diffusion strategy for UAV motion control.

In our UAV motion control problem, the CTA diffusion strategy, as shown in Figure 4, is also a two-step optimization process. The UAVs’ motion is not directly controlled by the UAV-UAV virtual force. Here we define a new parameter $\vec{FV_{i}\left[n\right]}$ as the local virtual force that controls $UAV_{i}$’s motion, which is initialized as 0 when n = 1 and updated by (15) and (16). $N_{i}$ is the set of neighbor UAVs of $UAV_{i}$.

In step 1, each UAV exchange $\vec{FV_{k}\left[n-1\right]}$ with neighbor UAVs and combine $\vec{FV_{k}\left[n-1\right]}$ that received from neighbor UAVs. Since $\vec{FV_{i}\left[n-1\right]}$ decides UAV’s motion, $\psi_{i}\left[n-1\right]$ can be treated as sum of movement trends of all neighbor UAVs. Step 1 can be expressed as

(15) $$\begin{array}{c} \psi_{i}\left[n-1\right]=\sum_{UAV_{k}\in N_{i}}\vec{FV_{k}\left[n-1\right]} \end{array}$$

In step 2, $\vec{Fv_{i,k}\left[n\right]}$ is the UAV-UAV force, which is defined as (7). $\vec{FV_{i}\left[n\right]}$ is the sum of two parts: the first part is the movement trends of all neighbor UAVs, says $\psi_{i}\left[n-1\right]$, and the second part is the resultant force of $\vec{Fv_{i,k}\left[n-1\right]}$ from neighbor UAVs for load balancing. Step 2 can be expressed as

(16) $$\begin{array}{c} \vec{FV_{i}\left[n\right]}=\psi_{i}\left[n-1\right]+\sum_{UAV_{k}\in N_{i}}\vec{Fv_{i,k}\left[n-1\right]} \end{array}$$

To be more specific, the CAT diffusion strategy is shown in Algorithm 1.
**Algorithm 1** Pseudocode for the CTA diffusion strategy1:For any $UAV_{i}$, $UAV_{k}$∈$N_{i}$, $\vec{FV_{i}\left[0\right]}$ is initialized as 0.2:at time *n*:3:Each UAV calculates its load through (8).4:$UAV_{i}$ calculates $\vec{Fv_{i,j}\left[n-1\right]}$ through (10).5:$UAV_{i}$ broadcasts it $\vec{FV_{k}\left[n-1\right]}$ to neighboring UAVs.6:$UAV_{i}$ calculates the sum of $\vec{FV_{k}\left[n-1\right]}$ ($UAV_{k}$∈$N_{i}$) through (15).7:$UAV_{i}$ calculates its UAV-UAV virtual force $\vec{FV_{i}\left[n\right]}$ through (16)

### 3.4. UAV-UE Virtual Force Calculation

Since the aim of $\vec{Fu_{i,j}\left[n\right]}$ is to improve the local performance, we will treat each UAV and its serving UEs as an individual system. Meanwhile, we assume that during UAV-UE virtual force optimization, the UEs’ association does not change. Thus, $B_{i,j}\left[n\right]$ in (6) is constant for the UAV-UE virtual force.

According to (13) and (14), calculating $\vec{FU_{i}\left[n\right]}$ is actually to find the optimal solution for each $\vec{Fu_{i,j}\left[n\right]}$ ($UE_{j}\in S_{i}$). Obviously, (12) is a non-convex problem for $x_{i}\left[n\right]$ and $y_{i}\left[n\right]$. To tackle the non-convexity, we will apply the successive convex optimization technique, such that (12) is approximated to be more tractable at a given local point [19,23]. By defining $\left\{{\overset{•}{x}}_{i}\left[n\right],{\overset{•}{y}}_{i}\left[n\right]\right\}$ as a given position of $UAV_{i}$ at time; meanwhile, introducing $d_{i,j}\left[n\right]$ in (1) as the variable, the problem can be approximated as

(17a) $$\begin{array}{cc} \; & \vec{Fu_{i,j}\left[n\right]}=Ku*\left(argmax_{\left(d_{i,j}\left[n\right]\right)}log\left(T_{i,j}\left[n\right]\right)\cdot \vec{D_{i,k}^{u}}\right) \end{array}$$

(17b) $$\begin{array}{cc} s.t. & \;T_{i,j}\left[n\right]=B_{i,j}\left[n\right]log\left(1+{\gamma \;}_{i,j}\left[n\right]\right) \end{array}$$

(17c) $$\begin{array}{cc} \; & {\;\gamma \;}_{i,j}\left[n\right]=\frac{p_{i}\left[n\right]g_{i,j}\left[n\right]}{\sum_{k\neq i}p_{k}\left[n\right]g_{k,j}\left[n\right]+\sigma^{2}} \end{array}$$

(17d) $$\begin{array}{cc} \; & \;g_{i,j}\left[n\right]=\rho_{0}d_{i,j}^{-2}\left[n\right]G_{i,j}\left({\overset{•}{x}}_{i}\left[n\right],{\overset{•}{y}}_{i}\left[n\right]\right) \end{array}$$

(17e) $$\begin{array}{cc} \; & \;G_{i,j}\left({\overset{•}{x}}_{i}\left[n\right],{\overset{•}{y}}_{i}\left[n\right]\right)=\left(\eta_{LoS}P_{i,j}^{LoS}\left[n\right]+\eta_{NLoS}P_{i,j}^{NLoS}\left[n\right]\right) \end{array}$$

(17f) $$\begin{array}{cc} \; & \;p_{i,j}^{LoS}\left[n\right]=\frac{1}{1+a\exp(-b\left(\frac{180}{\pi }tan^{-1}\left(\frac{H}{r_{i,j}\left[n\right]}-a\right)\right)} \end{array}$$

(17g) $$\begin{array}{cc} \; & \;r_{i,j}\left[n\right]=\sqrt{{\left({\overset{•}{x}}_{i}\left[n\right]-{\tilde{x}}_{j}\right)}^{2}+{\left({\overset{•}{y}}_{i}\left[n\right]-{\tilde{y}}_{j}\right)}^{2}} \end{array}$$

(17h) $$\begin{array}{cc} \; & \;\vec{D_{i,j}^{u}}=\frac{\left[\left({\tilde{x}}_{k}\left[n\right]-{\overset{•}{x}}_{i}\left[n\right]\right)\right],\left({\tilde{y}}_{k}\left[n\right]-{\overset{•}{y}}_{i}\left[n\right]\right)]}{\sqrt[{2}]{{\left({\tilde{x}}_{k}\left[n\right]-{\overset{•}{x}}_{i}\left[n\right]\right)}^{2}+{\left({\tilde{y}}_{k}\left[n\right]-{\overset{•}{y}}_{i}\left[n\right]\right)}^{2}}} \end{array}$$

In (17d), $g_{i,j}\left[n\right]$ is a concave function with respect to $d_{i,j}\left[n\right]$. For (17a), the second derivative of $log(T_{i,j}\left[n\right])$ with respect to $d_{i,j}\left[n\right]$ is

(18) $$\begin{array}{c} \frac{d_{2}\left(log\left(T_{i,j}\left[n\right]\right)\right)}{d{\left(d_{i,j}\left[n\right]\right)}^{2}}=\left(-2\overset{•}{\underset{i,j}{A}}\left[n\right]\right)B_{i,j}\left[n\right]\left[\frac{2{\overset{•}{A}}_{i,j}\left[n\right]d_{i,j}^{-6}\left[n\right]}{{\left(1+{\overset{•}{A}}_{i,j}\left[n\right]d_{i,j}^{-2}\left[n\right]\right)}^{2}}-\frac{3d_{i,j}^{-3}\left[n\right]}{1+\overset{•}{A}i,j\left[n\right]d_{i,j}^{-2}\left[n\right]}\right] \end{array}$$

where

(19) $$\begin{array}{c} {\overset{•}{A}}_{i,j}\left[n\right]=\frac{p_{i}\left[n\right]}{\sum_{k\neq i}p_{k}\left[n\right]g_{k,j}\left[n\right]+\sigma^{2}}\rho_{0}G_{i,j}\left({\overset{•}{x}}_{i}\left[n\right],{\overset{•}{y}}_{i}\left[n\right]\right) \end{array}$$

Note that *log*($T_{i,j}\left[n\right]$) is not concave with respect to $d_{i,j}\left[n\right]\in \left(-\infty ,+\infty \right)$. In our UAV deployment problem, $d_{i,j}\left[n\right]$ is limited by the location range of the UAVs and UEs. For any given location range, i.e., a 2000 m × 2000 m area, it can be simply proved by simulations that $\frac{d(\log\left(T_{i,j}\left[n\right]\right))}{d(d_{i,j}\left[n\right])}$ is positive and definite. Thus, in a real UAV deployment scenario, the approximated *log*($T_{i,j}\left[n\right]$) by successive convex approximation is a concave function. So the virtual force can be calculated by searching for the optimal solution of $argmax_{\left(d_{i,j}\left[n\right]\right)}$*log*($T_{i,j}\left[n\right]$) with its gradient as follows

(20) $$\begin{array}{c} \vec{Fu_{i,j}\left[n\right]}=Ku\times \left(\frac{d(\log\left(T_{i,j}\left[n\right]\right))}{d(d_{i,j}\left[n\right])}\cdot \vec{D_{i,k}^{u}}\right) \end{array}$$

### 3.5. UAV Motion Control Algorithm

The resultant force of $UAV_{i}$’s motion control can be calculated as

(21) $$\begin{array}{c} \vec{F_{i}\left[n\right]}=\vec{FV_{i}\left[n\right]}+\vec{FU_{i}\left[n\right]} \end{array}$$

The motion of $UAV_{i}$ is controlled by the resultant force $\vec{F_{i}\left[n\right]}$ iteratively. The maximum speed of a UAV is given by Vmax. The speed of a UAV should be an increasing function with respect to $\vec{F_{i}\left[n\right]}$. Meanwhile, the speed should be less sensitive to the increase in a large $\vec{F_{i}\left[n\right]}$ [5]. According to (14) in [5], we define the UAV moving velocity by

(22) $$\begin{array}{c} \vec{V_{i}\left[n\right]}=\arctan\left(\vec{F_{i}\left[n\right]}\right)\times \frac{2}{\pi }\times V_{\max} \end{array}$$

Therefore, the UAV motion control for load balancing is performed in three steps as below.

(1) Initialization: each UAV creates its neighboring UAV set according to neighbor UAVs’ distance [5].
(2) UAV motion control: UAVs’ motion is controlled iteratively. In each iteration, a UAV calculates FU by local load, neighbor UAVs’ load, and virtual force in a diffusion manner according to (8), (13), and (14). Then a UAV calculates the UAV-UE virtual force according to (11) and (18). Through (19), the resultant force is used for UAV motion control through (20).
(3) Stop condition: in an ideal condition, the UAVs stop when the virtual forces become zero. However, it will take too many steps to achieve perfect zero of the resultant force [5]. Thus, we assume that the optimization stops when all UAVs’ velocities are less than a threshold Vstop. To be more specific, the UAV motion control algorithm for load balancing is given in Algorithm 2.

Through the above three steps, UEs’ data rate fairness unitily (7) and the UAVs’ load balancing unility (9) can both be maximized. The performance is evaluated in the next section.
**Algorithm 2** Pseudocode for our algorithm1:For any $UAV_{i}$, $UAV_{k}$∈$N_{i}$2:Initialize $\vec{FV_{k}\left[0\right]}=0$, initialize $\vec{Fv_{i,k}\left[0\right]}$ by (10)3:**for** n > 0 **do**  UEs upload their data rate information to the serving UAV.4:UAV-UAV virtual force  $UAV_{i}$ broadcast its location.  $UAV_{i}$ creates its neighboring UAV set according to the received location.  $UAV_{k}$ shares $\vec{FV_{k}\left[n-1\right]}$ with its neighboring UAVs, (including $UAV_{i}$).  $UAV_{i}$ calculates $\psi_{i}\left[n-1\right]$ by (15).  $UAV_{i}$ updates $\vec{FV_{i}\left[n\right]}$ by (16).5:UAV-UE virtual force  $UAV_{i}$ calculates $\vec{Fu_{i,j}\left[n\right]}$, $\vec{FU_{i}\left[n\right]}$ by (20), (13), respectively.6:Resultant virtual force  $UAV_{i}$ calculate the resultant virtual force by (21).7:Motion Control8:Calculate the velocity $\vec{v_{i}\left[n\right]}$ by (22).9:**if** all $|\vec{v_{i}\left[n\right]}|$<$V_{stop},\left(i\in \left[1,M\right]\right)$10:      **breake**11:   **else do**12:      $\left\{\left[x_{i}\left[n+1\right],y_{i}\left[n+1\right]\right]\right\}=\left\{x_{i}\left[n\right],y_{i}\left[n\right]\right\}+\vec{v_{i}\left[n\right]}$13:   **end if**

## 4. Performance Evaluation

In this section, we conduct the simulations to evaluate the performance of the proposed UAV deployment algorithm. We will give the simulation results for UAVs’ load balancing and UEs’ data rate fairness.

### 4.1. Simulation Setup

We use Matlab (R2020a) as a simulator to implement the proposed algorithm. We use similar simulation settings as [5] did. As [5] did, we also take a set of bounded data to justify the effectiveness of the proposed algorithm on the UAVs’ load balancing and UEs’ data rate fairness. This is because this setting is similar to the real scenario. Generally, the area and number of UEs of disaster areas or hot spots are bounded.

Considering a 2000 m × 2000 m area, the UEs are randomly distributed in this area. UEs are not moving during the optimization. The number of UAVs and UEs are not fixed since we will discuss various scenarios in the following. The UAV motion control algorithm in [5] is the baseline for performance comparison.

We use the following metrics for performance evaluation.

(1) Number of UEs associated with each UAV. This is an intuitive performance that shows the UAVs’ load for each UAV.
(2) Fairness index of UAVs’ load.
(3) The CDF (Cumulative Distribution Function) of all UEs’ data rates.

System parameters are given in Table 1 unless otherwise specified.

### 4.2. UAVs’ Locations and UEs’ Association

**Figure 5:**
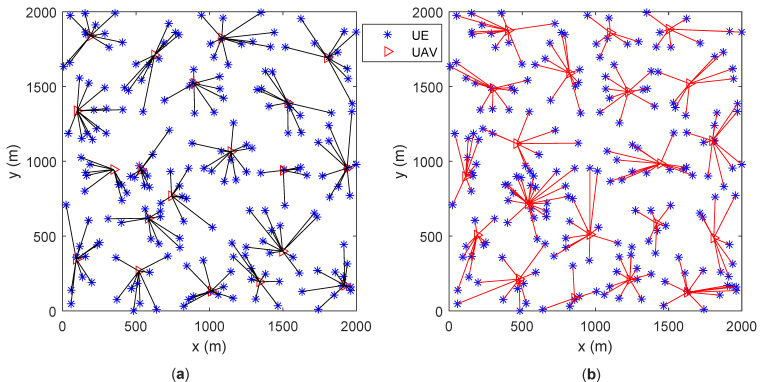
UAVs’ locations and UEs’ association (200 UEs). (**a**) proposed algorithm; (**b**) baseline algorithm [5].

**Figure 6:**
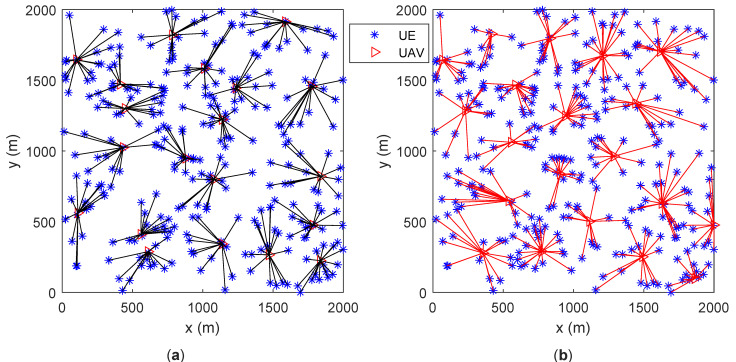
UAVs’ locations and UEs’ association (300 UEs). (**a**) proposed algorithm; (**b**) baseline algorithm [5].

Assume that UEs are randomly distributed in the area. The number of UAVs is 20. All UAVs’ locations are initialized to be in the center of the area. Sub figures (a) and (b) in both Figure 5 and Figure 6 give the UAVs’ locations as well as UEs’ association results after motion optimization by the proposed algorithm and the baseline algorithm in [5], respectively. The lines connecting UE and UAV represent that the UE is associated with the UAV. Figure 5 contains 200 UEs and Figure 6 contains 300 UEs.

In Figure 5a and Figure 6a, the number of UEs associated with each UAV is similar. In Figure 5b and Figure 6b, some of the UAVs server too many UEs or only a few UEs. Under various optimization metrics, the number of UEs served by a UAV is always an intuitive load performance indicator. It can be derived that the proposed algorithm achieves a more balanced load among all UAVs. The distance between UAVs is the main factor for motion control in the base line algorithm. Through factor, UAVs have similar coverage on the ground, which ignores that users may not be evenly distributed. The proposed algorithm optimizes UAVs’ motion by considering the load as well as the UEs’ distribution. Thus, the proposed algorithm outperforms the baseline algorithm.

**Figure 7:**
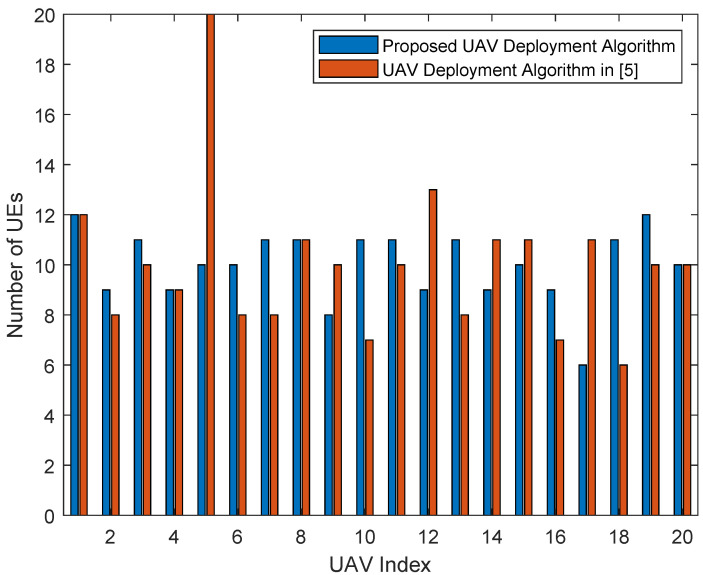
Number of UEs served by UAVs (200 UEs).

**Figure 8:**
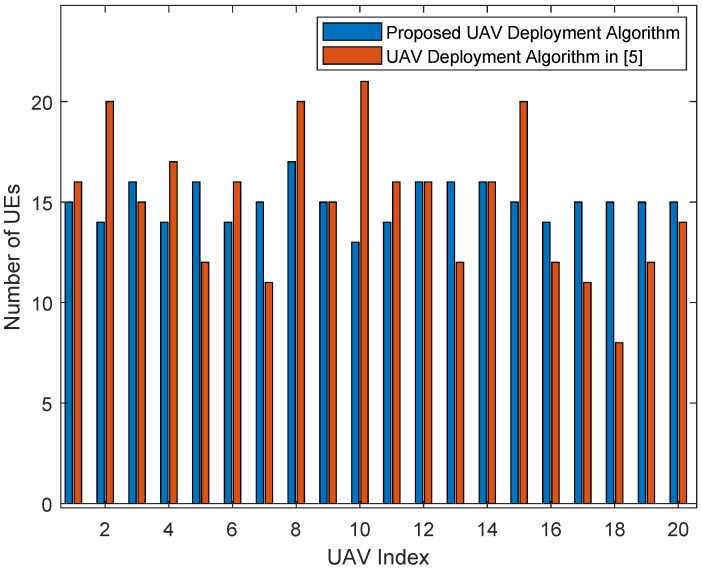
Number of UEs served by UAVs (300 UEs).

Figure 7 and Figure 8 are the bar graphs showing the number of UEs severed by UAVs, which corresponds to Figure 5 and Figure 6, respectively. The x-axis is the UAVs’ index and the y-axis is the number of UEs. It can be seen that the UEs’ distribution of the proposed algorithm is more even than the baseline algorithm.

**Figure 9:**
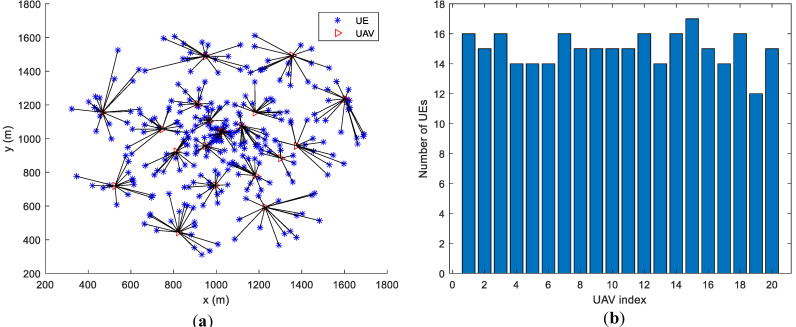
Load performance when UEs are distributed in a circular area with a radius of 800 m. (**a**) UEs’ Distribution and Associations. (**b**) Number of UEs of each UAV.

**Figure 10:**
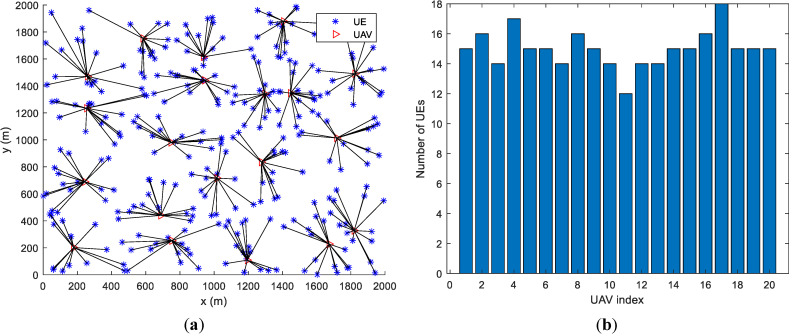
Load performance when UEs are distributed in a 2000 m × 2000 m square area. (**a**) UEs’ Distribution and Associations. (**b**) Number of UEs of each UAV.

**Figure 11:**
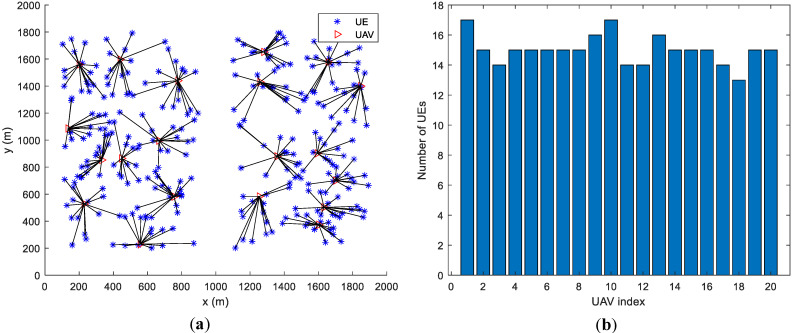
Load performance when UEs are distributed in two separate 800 m × 1600 m square areas. (**a**) UEs’ Distribution and Associations. (**b**) Number of UEs of each UAV.

By changing the UEs’ distribution, we found that the proposed algorithm achieves load balancing regardless of the UEs’ distribution. In Figure 9, Figure 10 and Figure 11 we assume that UEs are distributed in a circular area with a radius of 800 m, in a 2000 m × 2000 m square area and in two separate 800 m × 1600 m square areas, respectively. All UAVs are initialized at the center of the whole area. By the proposed algorithm, the numbers of UEs served by every UAV are very close, which means that the load balancing among the UAVs can be achieved regardless of the scenarios and UEs’ distributions.

### 4.3. Fairness Index of UAVs’ Load

**Figure 12:**
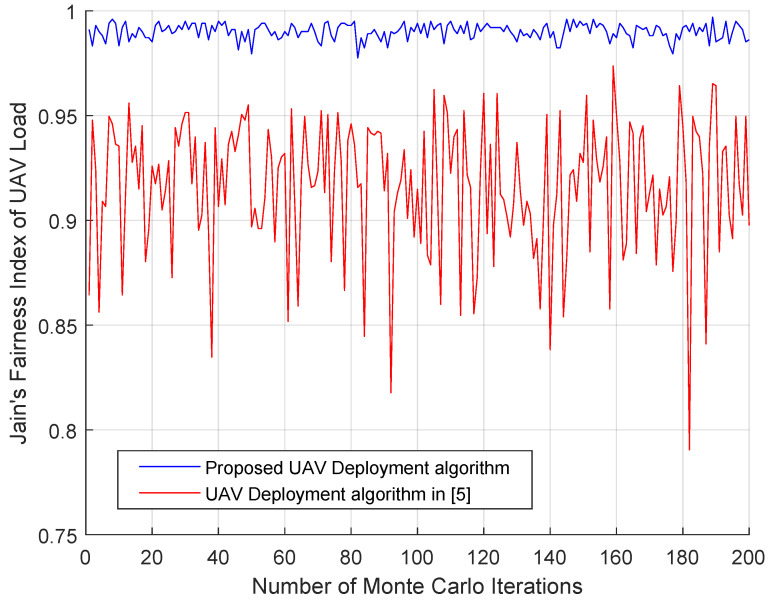
Jain’s fairness index of UAVs load.

This section gives the fairness index of UAVs’ load in 200 Monte-Carlo runs. The fairness index is to check the load distribution among UAVs, which is defined by Jain’s fairness index as

(23) $$\begin{array}{c} \psi =\frac{{\left(\sum_{i=1}^{M}N_{i}\left[n\right]\right)}^{2}}{\sum_{i=1}^{M}(N_{i}\left[n\right]{)}^{2}} \end{array}$$

where $N_{i}\left[n\right]$ is number of UEs severed by $UAV_{i}$. In each run, 200 UEs’ locations are initialized randomly and 20 UAVs are initialized at the center of the area. Figure 12 shows the simulation result of the fairness index, from which we can see that the proposed algorithm the fairness index is stable and larger than that of the baseline algorithm. The baseline algorithm achieves a large fairness index only when the UEs are evenly distributed in the area.

### 4.4. UEs’ Data Rate

**Figure 13:**
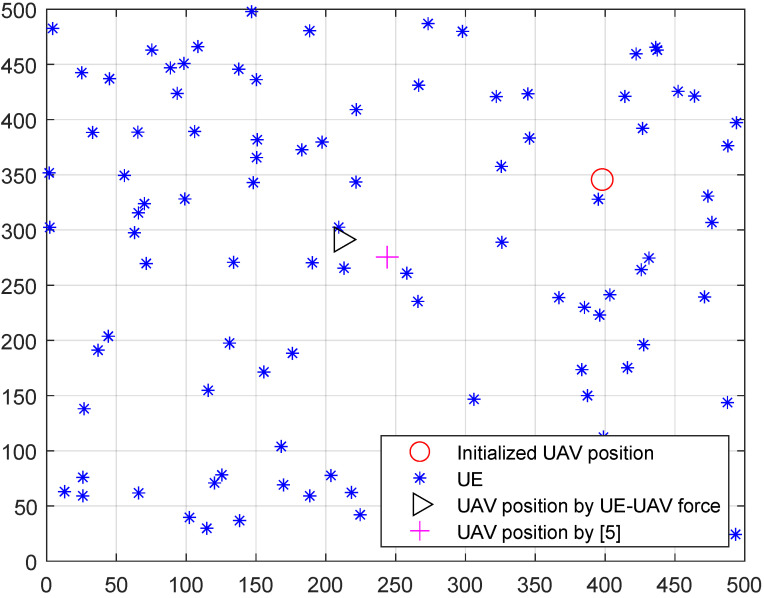
Single UAV deployment result comparison.

**Figure 14:**
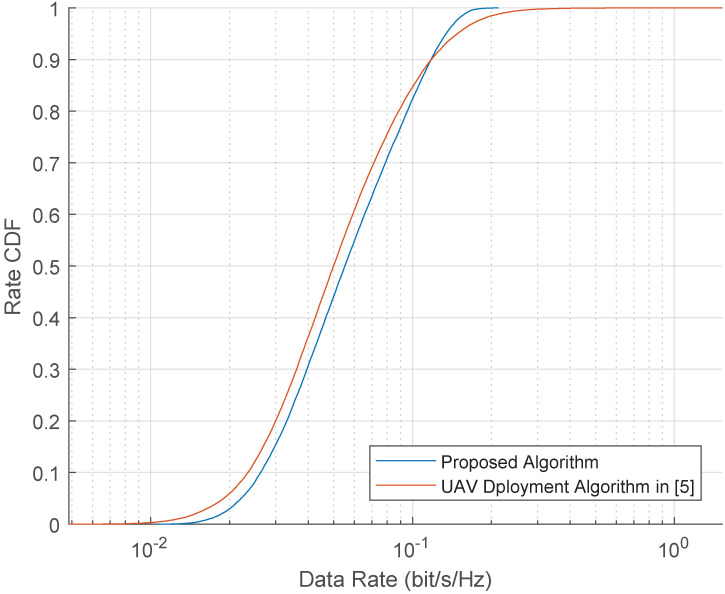
CDF of UEs’ data rate in 200 Monte Carlo runs.

The UEs’ data rate depends on the downlink channel state from UAV to UEs. In our problem, the location of the UAVs will decide the UEs’ data rate. To find out the intuitive effect of the proposed virtual force, we first compare the UAV deployment result by proposed virtual and baseline virtual force in [5] in a single UAV network, as shown in Figure 13. Considering a 500 m × 500 m area with 50 UEs, the UAV is initialized at a random location, which is shown as the circle in Figure 13. The UAV’s final positions after optimization by the proposed virtual force and baseline virtual force in [5] are represented by a triangle and a cross, respectively. The cross in Figure 13 is closer to the center of the whole area since the virtual force in [5] is defined by a first-order linear function of the distance between the UAV and UE. However, the data rate of a UE is a second-order non-linear function of distance according to (1)–(6). Thus, the baseline method can only achieve a near optimal for UEs’ data rate fairness. In our work, by constructing a new UE-UAV force as (14) based on data range, we can achieve a better fair UEs’ data rate result, which is shown in Figure 14.

Let us go back to the simulation setup in subsection A, the CDF of UEs’ data rate is shown in Figure 14. The available bandwidth of each UAV is normalized as one. Through 200 Monte Carlo runs, it can be derived that the proposed algorithm improves the performance of low data rate UE compared with the baseline algorithm, and the fairness among UEs is improved.

## 5. Discussions

In this paper, we show that the UAVs’ load balancing problem and the UEs’ data rate fairness in multi-UAV networks can be jointly optimized by UAVs’ movement control. Importantly, we find a unified performance metric for the above two problems. This joint optimization problem has not been widely studied yet in multi-UAV communication networks. We define two new virtual forces concerning the unified performance metric to control the UAVs’ movement. By adopting the diffusion strategy, we find a way to optimize the UAVs’ movement iteratively in a distributed manner. The simulation results confirm that:

(1) UAVs’ load balancing problem and the UEs’ data rate fairness can be optimized simultaneously.
(2) After 200 Monte-Carlo simulations, the fairness index on UAVs’ load of the proposed algorithm is always larger than 0.975. This performance of the baseline algorithm varies from 0.79 to 0.96. Compared with the virtual force defined by the locations of the UAVs and the UEs (baseline algorithm [5]), the virtual force defined by the utility function performs better on UAVs’ load balancing.
(3) Figure 9 and Figure 11 imply that the proposed algorithm achieves UAVs’ load balancing regardless of the UEs’ distribution.

Although there are important discoveries revealed by our studies, there are also limitations. In our scenario, the UEs are assumed to be static since the UAVs move much faster than the UEs. However, this assumption does not hold in a real multi-UAV communication network. We will study the dynamic scenario soon and we believe this is one important future direction of this topic.

## 6. Conclusions

This paper studies the joint UAV’s load balancing and UE’s data rate fairness optimization in multi-UAV communication networks. A unified utility is defined for the above two problems. With this unified utility, we then construct two virtual forces to control UAVs’ movement. Furthermore, we consider the CTA diffusion strategy and the successive convex optimization techniques for searching the optimal virtual forces for UAVs’ load balancing and UEs’ data rate fairness. Simulation results show that the proposed algorithm can achieve a better load balancing performance compared with the baseline algorithm. Through 200 Monte Carlo simulations and various UEs’ distribution settings, the load balancing performance is stable, which means that the performance is independent of UEs’ distribution. Meanwhile, the fairness of UEs’ data rate is improved. Next, we consider optimizing UAVs’ load balancing in dynamic UE scenarios and focus on UEs’ motion mode prediction.

## Figures and Tables

**Table 1:** System Parameters for Simulations.

Parameter	Symbol	Value
Height of UAVs	*H*	100 m
Communication range of UAVs	$R_{c}$	500 m
Tx power of UAV	*P*	20 dBm
Neighboring UAV distance Threshold	$R_{N}$	250 m
Carrier frequency of transmission channel	*f*	2 GHz
a		9.6
b		0.28
LoS additional pathloss	$\eta_{LoS}$	1 dB
NLoS additional pathloss	$\eta_{NLoS}$	20 dB
Maximum speed of UAV	$V_{max}$	10 m/s
UE-UAV force factor	$K_{u}$	1
UAV-UAV force factor	$K_{v}$	3

## Data Availability

Not applicable.

## Abbreviations

UAV	Unmanned Aerial Vehicle
BS	Base Station
UE	User Equipment
LoS	Line-of-Sight
NLoS	Non-Line-of-Sight
LMS	Least Mean Square
SINR	Signal to Interference Plus Noise Ratio
AWGN	Additive White Gaussian Noise
CTA	Combine-then-Adapt
CDF	Cumulative Distribution Function
FV	UAV-UAV virtual force
FU	UAV-UE virtual force
